# Synchronous Intestinal Tuberculosis and Ulcerative Colitis: A Diagnostic Challenge

**DOI:** 10.7759/cureus.88132

**Published:** 2025-07-17

**Authors:** Joana Cartucho, Bruno Bonito, B Pena, Catarina Lima Vieira, Carla Fernandes

**Affiliations:** 1 Internal Medicine, Unidade Local de Saúde do Arco Ribeirinho, Barreiro, PRT; 2 Anatomic Pathology, Unidade Local de Saúde do Arco Ribeirinho, Barreiro, PRT; 3 Gastroenterology, Unidade Local de Saúde do Arco Ribeirinho, Barreiro, PRT

**Keywords:** diagnostic challenge, immunocompromised host, inflammatory bowel disease, intestinal tuberculosis, ulcerative colitis

## Abstract

Intestinal tuberculosis (ITB) is a rare manifestation of extrapulmonary tuberculosis that often mimics inflammatory bowel diseases, particularly ulcerative colitis (UC), complicating the diagnostic process. We report the case of a 54-year-old woman with poorly controlled type 2 diabetes who presented with a five-month history of abdominal pain, diarrhea, bloating, fatigue, and low-grade fever. Initial imaging suggested features consistent with inflammatory bowel disease (IBD), and colonoscopy revealed mucosal ulcerations. Histopathological examination confirmed a diagnosis of ITB, and the patient was started on standard anti-tuberculous therapy. However, clinical improvement remained limited. A follow-up colonoscopy six months later demonstrated persistent inflammatory changes, raising suspicion of a coexisting UC. Initiation of mesalamine therapy resulted in significant symptom resolution. This case underscores the diagnostic challenge posed by overlapping gastrointestinal pathologies such as ITB and UC. Timely recognition of coexisting conditions is critical for guiding appropriate treatment and optimizing patient outcomes.

## Introduction

Intestinal tuberculosis (ITB) is an uncommon form of extrapulmonary tuberculosis (TB) caused by *Mycobacterium tuberculosis*, accounting for approximately 1-3% of all TB cases and up to 11% of extrapulmonary TB presentations [1]. While any segment of the gastrointestinal tract may be involved, the ileocecal region is most frequently affected. Although relatively rare, the incidence of ITB is rising, particularly in areas with high TB prevalence and among immunocompromised populations [1].

Ulcerative colitis (UC) is a chronic inflammatory bowel disease (IBD) primarily affecting the colon and rectum. It shares overlapping clinical, radiological, and endoscopic features with ITB, making accurate differentiation between the two conditions particularly challenging [2]. Several mechanisms may contribute to the susceptibility of UC patients to ITB, including chronic mucosal ulceration that facilitates mycobacterial invasion and immune dysregulation that compromises host defense, even in the absence of immunosuppressive therapy [2].

Here, we present the case of a 54-year-old woman with poorly controlled type 2 diabetes who was concurrently diagnosed with ITB and UC, highlighting the diagnostic complexity and therapeutic implications of coexisting gastrointestinal pathologies. Notably, the patient had no known epidemiological risk factors or exposure history for TB, underscoring the diagnostic challenge in non-endemic settings.

## Case presentation

A 54-year-old female with a nine-year history of poorly controlled type 2 diabetes presented to the emergency room with a five-month history of abdominal pain, bloating, and diarrhea, with four to five bowel movements per day, including nocturnal episodes and visible blood. She also reported fatigue, intermittent low-grade fever, and unintentional weight loss of 15 kg (approximately 28% of her baseline weight). She denied respiratory symptoms, night sweats, or other gastrointestinal manifestations. Despite symptom onset approximately five months before presentation, she did not seek medical attention until her symptoms worsened and fever developed.

There was no relevant family history, and the patient had no known exposure to TB. She had no history of smoking, alcohol use, intravenous drug use, or employment in healthcare settings.

On physical examination, her vital signs were within normal limits. The abdomen was soft, depressible, non-tender, and exhibited hyperactive bowel sounds. Her body mass index (BMI) was 14.3 kg/m². A capillary (fingerstick) blood glucose measurement indicated hyperglycemia at 352 mg/dL.

Initial blood tests revealed normocytic normochromic anemia, leukopenia, thrombocytosis, elevated C-reactive protein (256 mg/L), hyperglycemia, and hyponatremia (Table 1).

**Table 1:** Laboratory test results.

Parameter	Hospital admission	Hospital discharge	Gastro consultant – nine-month follow-up	Gastro consultant - 17-month follow-up	Gastro consultant- 30-month follow-up	Reference range
Hemoglobin (g/dL)	9.6	9.4	11.1	12.4	12.3	12.0–15.0
Hematocrit (%)	28.9	28	33.2	36.9	36.5	36–46
Mean corpuscular volume (fL)	85	85	80	85	92	83–101
Mean corpuscular hemoglobin (pg)	29	28	27	29	31	27–32
White blood cells (x10^9/L)	3.30	7.30	4.30	7.10	5.80	4–10
Neutrophils (x10^9/L)	0.8	4.3	1.8	4.7	3.9	2.1–7.5
Platelets (x10^9/L)	466	541	340	310	235	150–400
Lactate dehydrogenase (UI/L)	143	-	-	-	-	125-243
Urea (mg/dL)	12	27	15	29	30	10–50
Creatinine (mg/dL)	0.35	0.39	0.47	0.55	0.50	0.55–1.02
Sodium (mmol/L)	133	135	-	-	-	136-145
Potassium (mmol/L)	4.4	4.1	4.6	4.6	4.4	3.5–5.1
Chloride (mmol/L)	99	98	-	-	-	98-107
Aspartate aminotransferase (U/L)	10	13	19	12	21	<34
Alanine aminotransferase (U/L)	7	12	17	11	21	<38
C-reactive protein test (mg/L)	256	17	41	7	16	<5
Fecal calprotectin (µg/g)	-	-	752	308	218	<50
Iron (µg/dL)	-	-	22	115	-	50–170
Ferritin (ng/mL)	-	-	5.3	40.7	39	30–204

**Figure 1:**
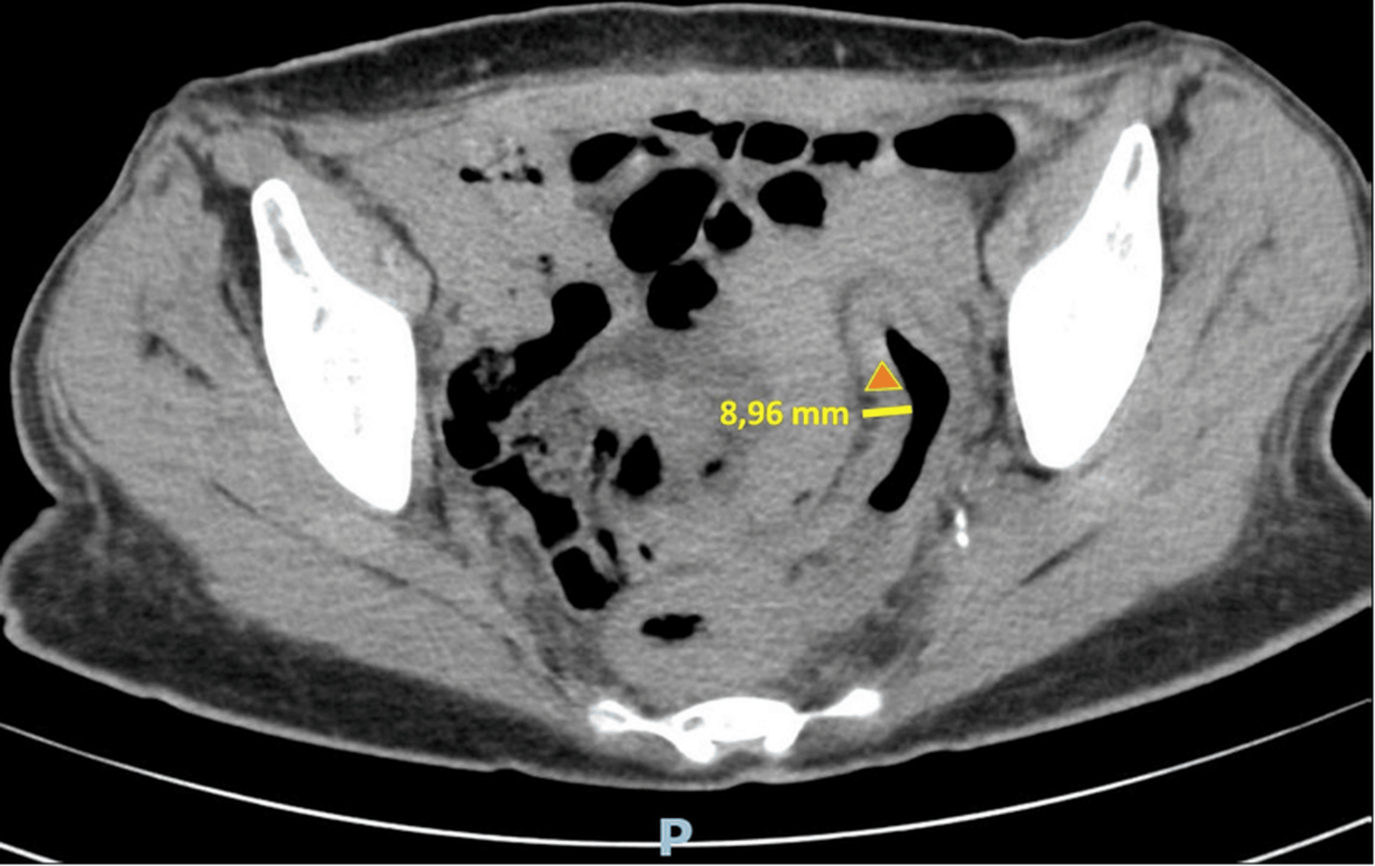
Pelvic CT (image 1 of 2) Axial CT image showing diffuse thickening of the colon (▲), compatible with active inflammation.

**Figure 2:**
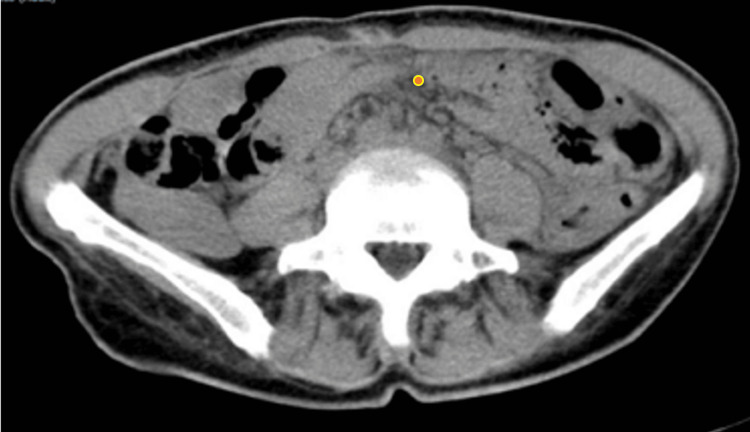
Pelvic CT (image 2 of 2) Axial CT image showing densification of all intraperitoneal adipose planes (●), suggestive of inflammatory fat stranding.

Chest radiography and non-contrast thoracic computed tomography (CT) were unremarkable. A non-contrast abdominal and pelvic CT revealed diffuse thickening of the entire colon (Figure 1), associated with densification of the intraperitoneal adipose tissue and the presence of several subcentimetric lymph nodes (Figure 2).

Given these findings, the patient was admitted for further diagnostic workup. As part of the etiological investigation for chronic diarrhea, thyroid function was found to be normal, and celiac serology (anti-transglutaminase IgA and IgG) was negative. Stool analysis showed no evidence of parasitic infection, bacterial pathogens, or *Clostridium difficile* toxin. Further testing revealed negative results for interferon-gamma release assay (IGRA), human immunodeficiency virus (HIV) types I and II, and herpes simplex virus (HSV). Serologies for Epstein-Barr virus (EBV) and cytomegalovirus (CMV) were consistent with past infections (IgG positive). Hepatitis B and C screening was also negative. Autoimmune testing showed positive anti-*Saccharomyces cerevisiae* antibodies (ASCA) IgG (59 RU/mL), with negative results for ASCA IgA, perinuclear anti-neutrophil cytoplasmic antibodies (pANCA), antinuclear antibodies (ANA), and anti-double-stranded DNA (anti-dsDNA) antibodies.

**Figure 3:**
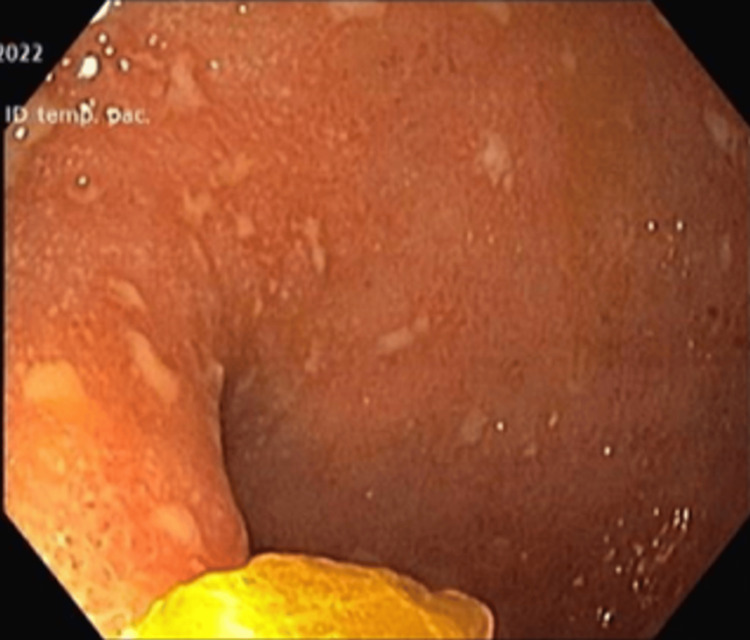
Colonoscopy prior to initiation of antituberculous therapy Endoscopic image showing diffuse mucosal inflammation with aphthous ulcers, loss of vascular pattern, friability, and superficial ulcerations suggestive of active colitis.

Upper endoscopy revealed no significant macroscopic abnormalities, although duodenal biopsies showed nonspecific chronic duodenitis. Colonoscopy was suboptimally prepared and interrupted at 30 cm due to mucosal erythema, aphthous ulcers, and pseudomembranes extending from the rectum (Mayo endoscopic subscore (MES) of 3) (Figure 3).

**Figure 4:**
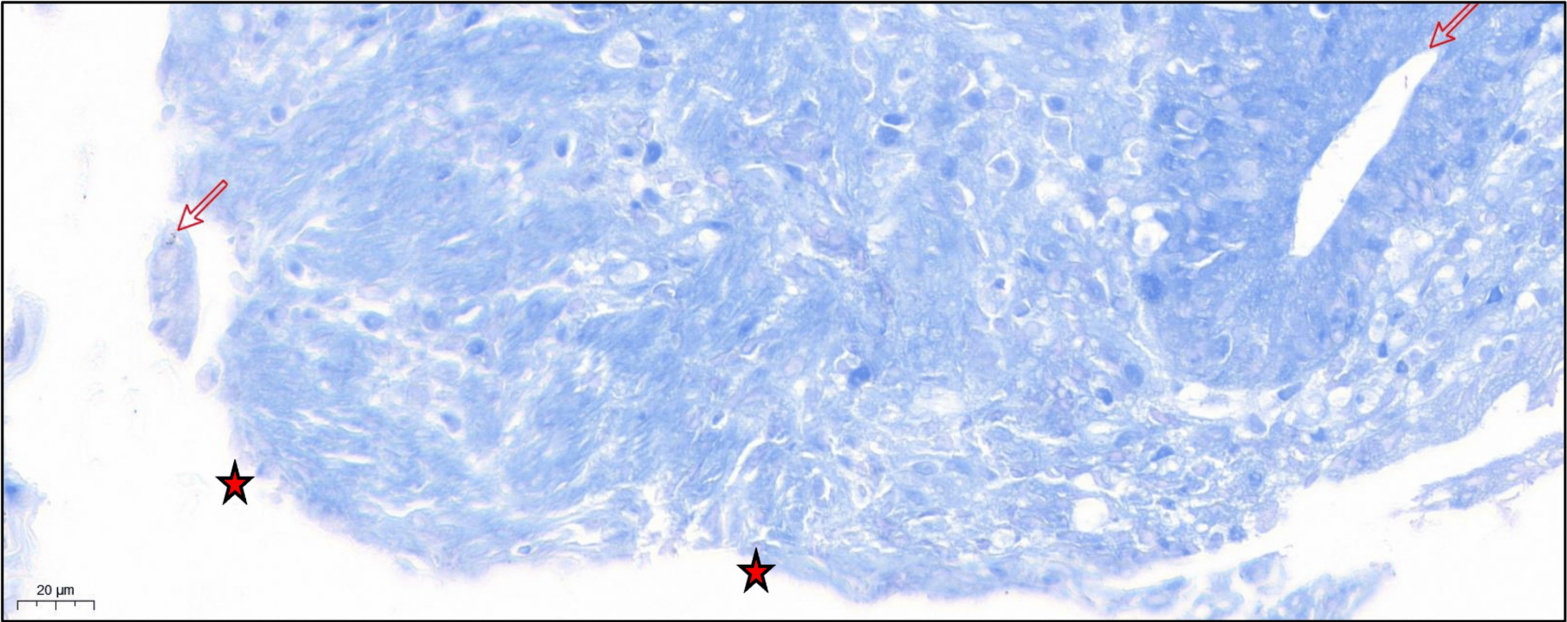
Biopsy prior to initiation of antituberculous therapy (image 1 of 2) Stain: Ziehl-Neelsen; magnification: 40×. Marked erosion of the superficial mucosa (★) with acid-fast bacilli (→) highlighted by Ziehl-Neelsen staining.

**Figure 5:**
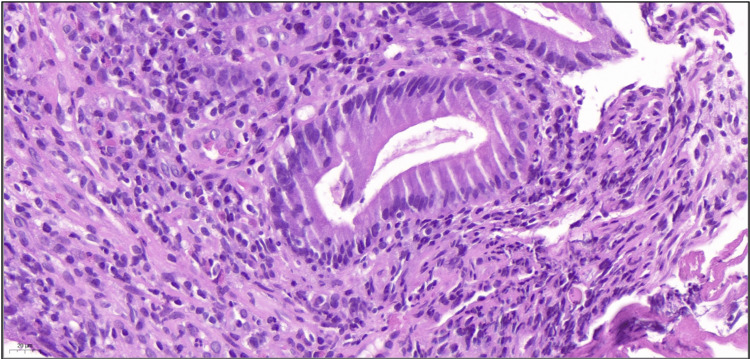
Biopsy prior to initiation of antituberculous therapy (image 2 of 2) Stain: Hematoxylin and eosin; magnification: 40×. The submucosa exhibits a moderate to dense inflammatory infiltrate, predominantly composed of lymphocytes and plasma cells, with scattered eosinophils and neutrophils. The latter infiltrate the mucosa and form small crypt microabscesses.

Colon biopsies revealed marked erosion of the superficial mucosa, with positive Ziehl-Neelsen staining identifying acid-fast bacilli (AFB) (Figure 4). The submucosa exhibited a moderate to dense inflammatory infiltrate, predominantly composed of lymphocytes and plasma cells, with scattered eosinophils and neutrophils forming crypt microabscesses (Figure 5). Immunohistochemical staining for CMV on colonic biopsy specimens was negative. Laboratory evaluation showed normal lactate dehydrogenase (LDH) levels and unremarkable serum protein electrophoresis, findings not supportive of lymphoma.

Empiric antibiotic therapy with metronidazole and ciprofloxacin was initiated (10 and seven days, respectively). Given the AFB-positive biopsy, anti-tuberculous therapy was commenced (two months of isoniazid, rifampicin, pyrazinamide, and ethambutol, followed by seven months of isoniazid and rifampicin). Clinical improvement followed, including a 5-kg weight gain and decreased bowel frequency to one to two bloodless, non-nocturnal stools daily.

**Figure 6:**
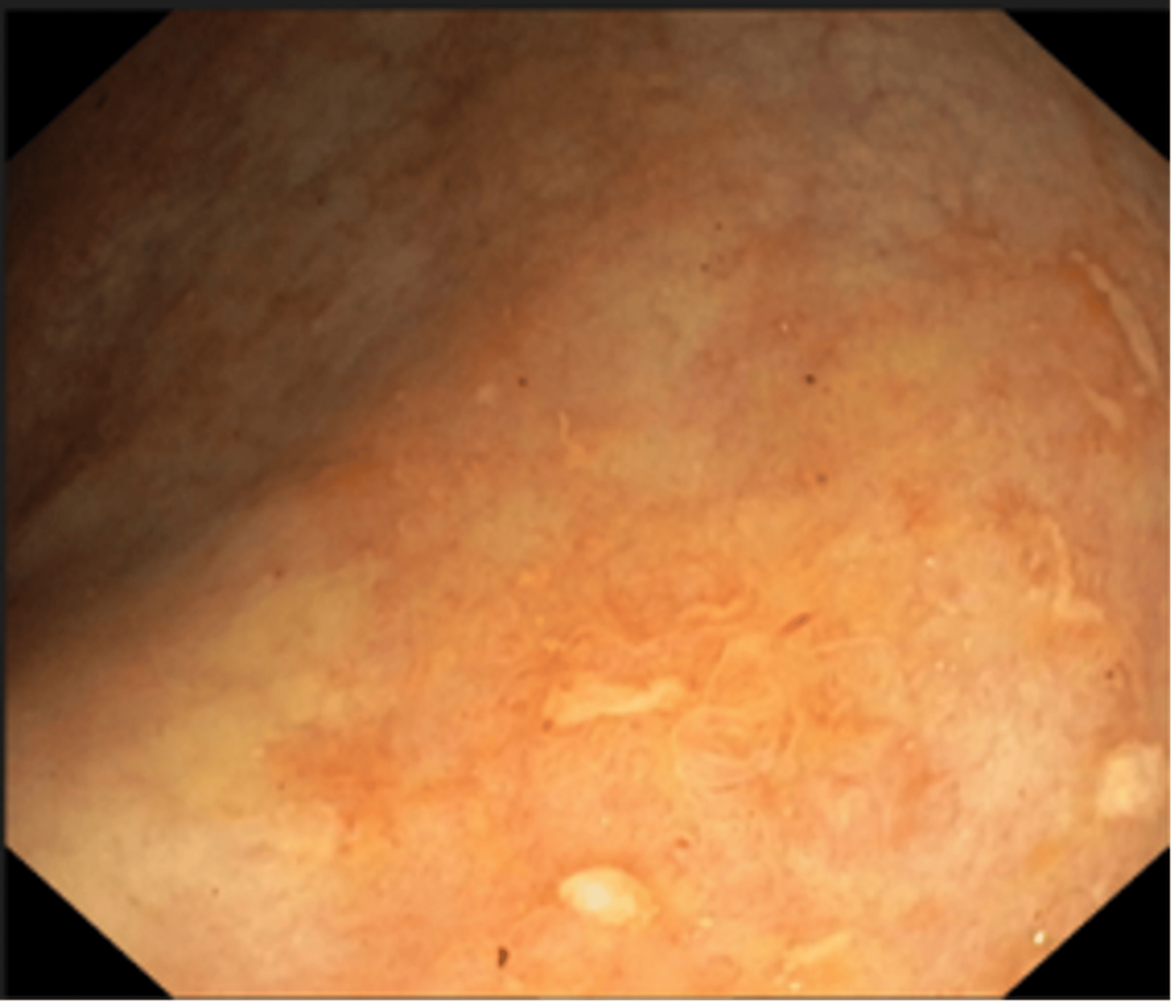
Colonoscopy following antituberculous therapy Segment showing mucosal re-epithelialization and patchy erythema with residual inflammatory changes.

**Figure 7:**
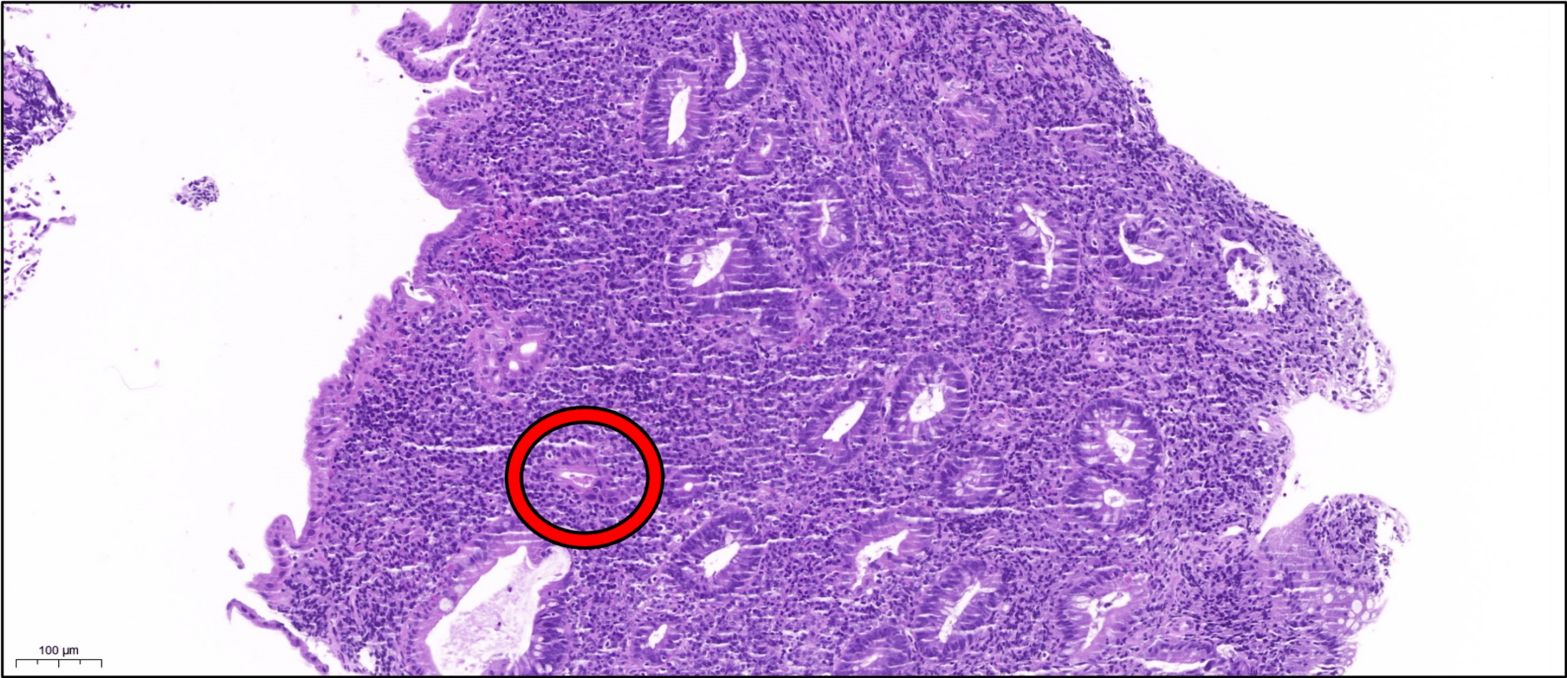
Biopsy following completion of antituberculous therapy Stain: Hematoxylin and eosin; magnification: 10×. Intestinal mucosal fragment showing architectural distortion with crypt branching and shortening. Superficial mucosal erosion is present. The submucosa displays a dense lymphoplasmacytic infiltrate rich in eosinophils and neutrophils. Neutrophils infiltrate the crypt epithelium (cryptitis), with partial crypt destruction observed in some areas (red circle).

After nine months of anti-tuberculous therapy, repeat colonoscopy demonstrated persistent microerosions and ulcerations in the rectosigmoid region, corresponding to an MES of 2 (Figure 6). Biopsies obtained during this procedure revealed architectural distortion and cryptitis with ongoing mucosal inflammation, suggestive of a chronic inflammatory process possibly consistent with ulcerative colitis (Figure 7). Given the negative result of polymerase chain reaction (PCR) testing for *M. tuberculosis*, anti-tuberculous therapy was discontinued.

In the weeks following treatment cessation, the patient reported resolution of diarrhea and a further 6 kg weight gain. However, she continued to experience occasional abdominal pain, myalgias, arthralgias, and low-grade fever. Considering the persistence of symptoms and prior histological evidence of chronic inflammation, a diagnosis consistent with ulcerative colitis was made. Oral mesalamine was initiated (4 g/day for induction, tapered to 2 g/day for maintenance), resulting in sustained clinical and biochemical improvement. Follow-up assessments confirmed symptomatic remission, normalization of inflammatory markers (C-reactive protein [CRP] and fecal calprotectin), and recovery of BMI to 18.44 kg/m² (Table 1).

## Discussion

ITB is more frequently encountered in developing countries, representing 1-3% of all TB cases and up to 11 % of extrapulmonary presentations [1]. However, its incidence has also increased in developed regions, particularly among immunocompromised individuals [1].

*M. tuberculosis* may affect any segment of the gastrointestinal tract, but the ileocecal region is most commonly involved due to its rich lymphoid tissue and relative stasis of intestinal contents [3]. The clinical presentation of ITB is often nonspecific and overlaps with other gastrointestinal diseases, particularly UC. Symptoms such as abdominal pain, diarrhea, weight loss, and fever are frequently observed, which may lead to diagnostic delays [4].

The diagnostic evaluation of ITB involves histopathological, microbiological, molecular, and radiologic approaches. Although caseating granulomas are suggestive, they are not always present. Ziehl-Neelsen staining and culture may support the diagnosis, although sensitivity is limited. Molecular tests such as PCR and GeneXpert MTB/RIF enhance diagnostic accuracy. Imaging studies may demonstrate concentric bowel wall thickening, and colonoscopy often reveals ulcerated or nodular mucosa [5]. It is crucial to note that the patient had no exposure to TB-endemic regions; poorly controlled diabetes was the only identifiable risk factor at admission, heightening diagnostic complexity.

Treatment for ITB employs a two-phase antituberculous regimen: an initial two-month intensive phase (isoniazid, rifampicin, pyrazinamide, ethambutol), followed by four to six months of continuation therapy (isoniazid, rifampicin) [6]. When administered early, this approach generally yields favorable clinical and mucosal outcomes [6].

Despite the standard treatment, our patient demonstrated persistent erythema and mucosal ulcerations at follow-up colonoscopy, consistent with an MES of 2. Histological examination confirmed ongoing cryptitis and mucosal architectural distortion, while polymerase chain reaction (PCR) testing for *M. tuberculosis* was negative. Based on these findings, antituberculous therapy was discontinued.

The persistence of inflammation following resolution of the infectious component raised the suspicion of underlying IBD. Although anti-*Saccharomyces cerevisiae* antibodies (ASCA) IgG positivity may be associated with Crohn’s disease (CD), the diffuse and continuous lesion distribution, rectal involvement, and absence of granulomas favored a diagnosis of UC over CD [6]. A comparative summary of distinguishing features is presented in Table 2.

**Table 2:** Comparative features between ulcerative colitis and Crohn's disease ASCA = anti‑*Saccharomyces cerevisiae *antibodies; pANCA = perinuclear anti‑neutrophil cytoplasmic antibodies. Source: Data adapted from Ahmed et al. [2] and Harbord et al. [7].

Characteristic	Ulcerative colitis	Crohn's disease
Location	Rectum and colon (continuous)	Entire gastrointestinal tract (skip lesions)
Inflammation	Mucosal and submucosal	Transmural
Endoscopic findings	Continuous lesions, pseudopolyps	Cobblestone appearance, aphthous ulcers, strictures
Histology	Crypt abscesses, mucosal ulceration	Granulomas, transmural lymphoid aggregates
Serology	pANCA+	ASCA+

Given the clinical and endoscopic findings suggestive of UC, oral mesalamine therapy was initiated. Current guidelines recommend 5-aminosalicylic acid (5-ASA) agents as first-line treatment for mild-to-moderate UC, and mesalamine is associated with a low risk of TB reactivation [7,8]. While combined oral and rectal formulations are often used for extensive disease [8], monotherapy led to symptomatic and biochemical remission in this patient, including normalization of CRP, fecal calprotectin, and recovery of BMI.

Mesalamine maintenance therapy was thus considered appropriate, offering durable remission without the need for immunosuppressive agents. The introduction of immunomodulators or biologics should be reserved for UC cases unresponsive to mesalamine or with high-risk features, with strict TB surveillance protocols in place [9].

## Conclusions

ITB remains an important differential diagnosis, particularly in regions with high TB prevalence and among immunocompromised individuals. Its nonspecific clinical presentation and significant overlap with other gastrointestinal disorders, such as IBD, underscore the need for thorough diagnostic evaluation. In patients with UC, factors such as mucosal barrier disruption, immune dysregulation, and the use of immunosuppressive therapies may increase susceptibility to ITB. Clinicians should maintain a high index of suspicion when UC patients exhibit atypical clinical features or fail to respond to standard therapy. Early diagnosis and appropriate management are critical to prevent complications and improve outcomes.
